# Progress in Medicine

**Published:** 1899-05-20

**Authors:** 


### PROGRESS IN MEDICINE.

### DISEASES OF THE NERVOUS SYSTEM.

(*Continued from page* 113.)

**Experimental Tabes.**—Edinger and Helbing[14] have recently reported the results of attempts at the experimental production of degeneration of the posterior columns. The animals, rats, were put into a revolving cage so that they were forced to undergo excessive muscular exertion for long periods. Degenerative changes were found in the posterior columns, most marked in the lumbar region, and, in severe cases, in the antero-lateral tracts. The external band of the posterior columns suffered more than any other part, and, as in tabes, the ventral part of the posterior columns escaped. If the animal were previously rendered anæmic by means of pyrodin (which of itself does not affect the cord) the degenerative changes were produced more rapidly. Thus, abnormally prolonged exertion in rats produces degeneration of the posterior columns precisely similar to that which occurs in cases of tabes in man, and if there be predisposing causes, such as anæmia, the same effect is more readily produced.

**Tabes and Syphilis.**—The statistical statements of Fournier and Erb in support of the theory that syphilis is a predisposing cause of tabes have often been questioned; though, insomuch as tabes is not a syphilitic process from an anatomical point of view, this is the only method available. Kuhn[15] has reinvestigated the question by comparing the histories of tabetic and non-tabetic patients from the neurological service of the Charité in Berlin for a series of years. He found that of the tabetic men 38·2 per cent. gave a conclusive history of syphilis; while of the men with other nervous diseases only 7·75 per cent. did so. The non-tabetic men gave a history of chancre with symptoms following which were suspicious of syphilis in 1·5 per cent.; while the tabetics gave such a history in 8·1 per cent. Of the tabetics, 21·3 per cent., while denying the primary lesion, had suffered from such symptoms as would tend to a diagnosis of syphilis; whilst, of the other patients, only 4·5 had had such symptoms. Finally, in only 11 per cent. of tabetics was there no evidence whatever of venereal disease; while of non-tabetics, 67·5 per cent. had this negative history. The results with the women were practically the same. The above paper thus adduces indisputable proof of the important *rôle* played by syphilis in the etiology of tabes. With an unmistakable history of syphilis more than five times as frequent, and no venereal history whatever four times less often, in tabetics as compared with non-tabetics, only one conclusion is admissible.

**Alcoholic Paralysis.**—Gaucher[16] draws attention to the great importance of preventing permanent deformity in cases of alcoholic paralysis. In the large majority of cases permanent drop-foot and other deformities are due to want of sufficient care on the part of the medical attendant. In all cases of alcoholic paralysis the antero-external group of muscles of the legs are early affected, allowing of drop-foot with internal rotation, and the pressure of the bed-coverings tends to aggravate this pernicious position. A rigid apparatus to prevent this deformity should be avoided, as pressure lesions may be set up owing to the low trophic condition of the feet. A simple wooden stand, thickly padded, may be safely used to maintain the foot at right angles to the leg, and a cradle may be used to support the bed clothes. As soon as the patient is able to bear it, passive movements and massage should be regularly employed to keep the mobility of the joints in a healthy condition and to promote the nutrition of the wasted muscles. The application of the galvanic current may also hasten recovery of power in the paralysed muscles.

**Polymyositis.**—Gowers[17] records a case of this rare disease, which consists in the simultaneous inflammation of many muscles, and of some nerves, symmetrical in distribution. The disease is met with almost exclusively as the result of cold. It seems to be a peculiar variety of the rheumatic poison produced in specially susceptible individuals rendered such by some influence which depresses the general health. The affection presents the general distribution of polyneuritis as far as preponderant loss of power is concerned, but with a far wider implication of the muscles of which many suffer that escape in ordinary polyneuritis. They are at first very tender, and afterwards undergo hardening and contraction which may be extreme in degree, and after a time insuperable. The patient was a woman with a family history of gout and rheumatism, and the affection slowly came on after constant exposure to cold, with pains in the back, then in hands and ankles, followed by increasing stiffness, weakness, and contraction of the muscles. On admission the patient was helpless. The facial muscles acted slowly and imperfectly, apparently from stiffness. There was a rigid contracture of all the muscles of the neck, which was quite stiff on the trunk. And the trunk was also stiff, so that the patient had to be moved in the bed as one solid piece. The arms were adducted to the sides of the trunk, flexed at the elbows, and the fingers were flexed. There was firm contraction of the flexors of the hips, knees, and of the calf muscles. Voluntary movement was very slight. All the muscles reacted very slightly to strong electrical stimulation, the faradic shock, or the interrupted voltaic current. The response to the latter was normal as far as the poles were concerned, and it had the sharp quick character characteristic of stimulation of intramuscular nerves. Sensibility of the skin was everywhere intact. The deep reflexes could not be anywhere obtained. There was no affection of the sphincters. The muscles were wasted, but extremely hard and firm. Partial recovery was brought about by passive extension, massage, and electricity.

**Erythromelalgia,** or red neuralgia, was first described in 1872 by Weir Mitchell, and the same physician, in conjunction with Dr. Spiller,[18] has recently published the following important case of the disease, the only one in which microscopical examination of affected parts has been made, and peripheral disease of arteries and nerves found. A man aged 61 years, in fair health, began to have limited areas of severe pain in the right foot, with tenderness and flushing when the limb hung down. While the fourth and fifth toes were thus affected, a small, deep, painful ulcer appeared on the outside of the fourth toe and healed slowly. After three months these toes seemed nearly well, and the second and third were attacked, and as these improved the great toe became affected. As usual, cold and elevation relieved the pain, heat and depression made it worse; walking was unendurable. The great toe was slightly red but not swollen. After many remedies had been tried in vain, the toe and half the metatarsal were amputated. The wound healed slowly with some sloughing. After the operation the patient had pain in the ball of the foot and some reddening of the toes, but he was better. The nerves of the great toe were intensely degenerated and showed scarcely any nerve fibres, consisting almost entirely of connective tissue. The media and intima of good-sized vessels were greatly thickened, and in some places the lumen was nearly obliterated. Weir Mitchell formerly held that erythromelalgia may be due to spinal disorder, but more recently has been inclined to attribute it to neuritis. The writers conclude that in their case the symptoms were due to peripheral neuritis, but are unable to say whether the arteriosclerosis or the neuritis in their case was the primary lesion. Auerbach has published a case of erythromelalgia in which the upper sacral and lower lumbar posterior roots were much degenerated, so that it would appear that involvement of sensory fibres anywhere between the cord and the periphery, and possibly within the cord, is capable of causing this disease.

[14] Edinger and Helbing, Trans. of 16th Congress of Exper. Med. [15] Kuhn, Archiv. für Psych. und Nerv., 1898. [16] Gaucher, Thèse de Paris, 1898. [17] Gowers, Brit. Med. Jour., Jan. 14, 1899. [18] Weir Mitchell and Spiller, Amer. Jour. Med. Sci., Jan., 1899.

---

### DISEASES OF THE STOMACH.

**Methods of Examination.**—Douglas[1] applies the chemical tests in the following order: (*a*) Litmus reaction; (*b*) change of Congo-red paper if H.Cl. or free organic acids be present; (*c*) calculation of total acidity given by the number of cubic centimetres of decinormal solution of NaH.O. required to neutralise 10 c.c. of the stomach contents; (*d*) determination of the presence of free H.Cl. by Gunzberg's test, or the cheaper "Tropœolin OO"—he considers that the continued absence of H.Cl. is at least a strong corroborative point in favour of the existence of cancer; (*e*) lactic acid, shown by the perchloride of iron test, though normally present at the beginning of digestion of starchy matters, disappears later on unless dilatation exists, or in some cases of cancer; (*f*) butyric and kindred acids show bacterial fermentation, and are found by shaking up with ether, in which they are soluble; (*g*) peptones, shown best by the biuret reaction, are rarely absent—sugar, starch, blood, and bile may be sought for by the ordinary tests, while pepsin is best shown by its decolourising fibrin stained with carmine; and (*h*) sarcinæ and fragments of tissue are finally sought for by the microscope. These tests can be all carried out in an hour, and afford valuable information. Bidwell[2] performs Gunzberg's test by dissolving 30 grains of phloroglucin and 15 of vanilin in an ounce of absolute alcohol. A drop of this is allowed to touch one of the stomach filtrate on a slip of china which is warmed. A bright-red tint appears if H.Cl. is present. Stern[3] insists on the value of noting the visible outlines of the stomach. Thus in gastroptosis the smaller curvature can be clearly seen if the patient lie horizontally in a good light. Rosenfeld[4] introduces a soft tube weighted with shot into the stomach, and then examines by the Röntgen rays, which show the shot marking the deepest part of the stomach. However, as Boardman Reed points out, the lower margin of the organ can be mapped out by making the patient drink two glasses of water, and then percussing first in the recumbent, and next in the upright posture. A marked area of dulness will be formed in the latter position over the lower margin. In another paper[5] on the value of washing out the stomach, he lays down that the stomach tube is more valuable for diagnosis than for treatment, and he cautions us against any long continued or too frequent use of it, quoting from Ewald that "when it does good, it does good very soon." If it brings up a large amount of mucus, we may suspect gastric catarrh, and in those cases lavage will be of great use, but we must guard against this diagnosis where the mucus has been swallowed and comes from the throat. Lavage, again, is very valuable in gastrectasis of every kind, as a palliative in the cases due to organic causes, and as an aid to cure in atonic ones. In bad gastric catarrh the stomach may at first be washed out every day, reducing this to twice a week, and after two months in bad cases to once a week. The best time is before breakfast, since less of the partially digested food is then removed to the loss of the patient. Einhorn[6] has invented a powder blower for the stomach. This must be used when the organ is empty after lavage. For gastric ulcer he gives in this way bismuth, for hæmorrhage antipyrine, for gastralgia orthoform, and for erosion protargol. By means of the Röntgen rays he has been able to demonstrate the diffusion of bismuth in this manner over the stomach wall. However, the real value of the treatment will have to be shown by the results, as the method is quite new. Turck[7] writes of the uses of his gyromele, and claims that by it the exact outline of the stomach and its distensibility can be made out without danger. Moreover, as its use stimulates the stomach just as the entrance of food does, the secretions of the organ are tested by its use if it be left in the stomach for 15 minutes, which many patients bear easily. It also removes mucus, and by it drugs can be applied to any part of the stomach. By means of a soft rubber bag at the end of a tube he has also been able to send a continuous stream of hot water through the stomach and out again in cases of shock; and at Edinburgh he demonstrated a method of treatment which depended on the introduction of a current of hot air into the organ and its rapid return. He thus produced a rapid dilatation and contraction of the walls, and obtained by this exercise a marked increase of their muscular power. Bensley[8] regards the pyloric gland cells of man as allied to certain glands in the fundus of other mammals which have been little noticed. It appears that certain parts only of these latter glands contain cells which are the homologues of the human pyloric glands, the rest of the gland being occupied only in the secretion of mucus. Roux and Balthazer[9] have examined the motor functions of the stomach by the aid of the Röntgen rays, and find that liquids begin to be evacuated in two or three minutes, while solids remain for some hours. Pure water was removed within ten minutes.

**Ulcer.**—Fremont[10] regards hyperacidity as the chief cause. To effect a cure we have to protect the ulcer and to neutralise the acidity as completely as possible. Now, milk fixes, and alkalies neutralise the H.Cl. if given frequently enough. Thus he orders an ounce of warm milk every half hour for 20 hours, to which magnesia, bismuth, &c., have been added. This may be given every hour only during sleep, and increased if the pain is not relieved. Soupault[11] recommends sodium chlorate for hyperchlorydria, whether ulceration is present or not. Two drachms are given daily in divided doses as far as possible from meal times. B. Tripier[12] argues that ulceration arises from primary inflammatory changes in the arteries, and recurrent hæmorrhages are due to a continuation of the inflammation in and around the vessels. Thus bleeding may take place without an ulcer, from rupture of a vessel. As we have before noticed, he recommends enemata of water at about 120 deg. Fah., with rectal feeding three times a day, or oftener, on the first appearance of hæmorrhage. Dominicis[13] shows by experiment that moderate fasting enables the organism to withstand the attacks of micro-organisms better than a full supply of food. Thus in pneumonia the virulence of the attack depends largely on the condition of the digestive tract.

**Minute Erosion of Gastric Arteries** with hæmorrhage has been discussed by T. Lindsay Steven.[14] He records two cases in which superficial abrasions with very small openings into arteries were present without any ulcer of the ordinary type. Both cases were fatal, one being in a woman and one in a man. Dieulafoy and several other authors have described such findings, the former giving seven cases where bleeding took place without an ulcer, cancer, or hyperæmia. Steven notices the profuse character of the hæmorrhage, and the comparative absence of the usual symptoms of gastric ulcer. Possibly the lesion is the earliest stage of ulceration. As to treatment medical measures may or may not stop the bleeding, but if surgical intervention is attempted we have to remember the difficulty of finding the bleeding point. In another paper[15] the author discusses the quickness with which general peritonitis follows perforation. Death in his two cases took place within 26 hours after complete perforation, so that surgical help, to be of use, must be given at a very early period.

**Varicose Veins in the Stomach** cannot be diagnosed clinically, but may occur in cirrhosis. Letulle[16] records two cases of fatal hæmorrhage thus produced. The veins undergo inflammation and pouches are developed; finally the mucous membrane which has been fixed to them becomes ulcerated, and the veins are opened. Le Wald[17] records a case of ulceration into the splenic artery with fatal hæmorrhage. A previous attack had occurred two years before, and a healed duodenal ulcer was also found in the same patient.

**Syphilitic Ulceration** of the stomach is rarely noticed in this country, though Fournier described two well-marked cases with hæmorrhage which readily yielded to specific treatment. W. A. Mackay[18] mentions an instance in a young man where the bleeding quickly ceased when the syphilitic origin was detected; and another where an almost moribund patient was saved by the use of mercury and iodides. He notices that bichromate of potash recently advocated in ulcer was largely used for syphilis in former days. S. Flexner[19] refers to some 22 recorded cases, and adds one of his own in a man aged 52. Perforation occurred, and syphilitic lesions were found in the liver, as well as in the stomach. He regards the ulcer as due to a specific necrosis of the mucous membrane, and not to the breaking down of a gumma, which seems to have been the cause in most of the older cases. Both hereditary and acquired syphilis may, it seems, present this complication. Potassium iodide seems, according to F. H. Simpson,[20] to be at times a cause of gastric ulcer, and at others a means of cure. Thus Neumann, in Vienna, recently showed some small ulcers of the stomach like those on the skin, which followed a long course of iodide in the case of a woman suffering from nephritis; while Dieulafoy speaks of its value in instances such as those described above by Mackay.

[1] Glasgow Med. Jour., Dec. [2] The Hospital, April 1. [3] Brit. Med. Jour., Feb. 11. [4] Brit. Med. Jour., Mar. 3. [5] Inter. Med. Mag., Nov. [6] New York Med. Jour., April 1. [7] Lancet, Jan. 28. [8] Lancet, Jan. 21. [9] Inter. Med. Jour., Jan. [10] Amer. Jour. Med. Sci., Nov. [11] Ibid. [12] Brit. Med. Jour., Jan. 21. [13] Ibid. [14] Glasgow Med. Jour., Jan. [15] Glasgow Med. Jour. Feb. [16] Brit. Med. Jour., Dec. 31. [17] Med. Rec., Dec. 17. [18] Lancet, Dec. 24. [19] Amer. Jour. Med. Sci., Oct. [20] Treatment, April 13.

